# Enhancing public health in developing nations through smartphone-based motor assessment

**DOI:** 10.3389/fdgth.2024.1345562

**Published:** 2024-05-21

**Authors:** Givago Silva Souza, Brena Karoline Ataíde Furtado, Edilson Brabo Almeida, Bianca Callegari, Maria da Conceição Nascimento Pinheiro

**Affiliations:** ^1^ Núcleo de Medicina Tropical, Universidade Federal do Pará, Belém, Brazil; ^2^ Instituto de Ciências Biológicas, Universidade Federal do Pará, Belém, Brazil; ^3^ Instituto de Ciências da Saúde, Universidade Federal do Pará, Belém, Brazil

**Keywords:** digital health, mHealth, smartphone, motor assessment, public health

## Abstract

Several protocols for motor assessment have been validated for use on smartphones and could be employed by public healthcare systems to monitor motor functional losses in populations, particularly those with lower income levels. In addition to being cost-effective and widely distributed across populations of varying income levels, the use of smartphones in motor assessment offers a range of advantages that could be leveraged by governments, especially in developing and poorer countries. Some topics related to potential interventions should be considered by healthcare managers before initiating the implementation of such a digital intervention.

## Introduction

Considering continual technological advancements and escalating demands within the public health sector, particularly in developing nations, it is imperative to carefully consider fundamental issues when advocating for digital health solutions as complementary tools in population healthcare. The World Health Organization (WHO), leveraging its extensive expertise amassed over several decades, has systematically reviewed evidence associated with digital technologies.

WHO presented a global strategy for advancing digital health for the years 2020–2025, anticipating the creation of digital solutions for the monitoring and surveillance of individuals' health in a significant number of countries (1). These strategies are grounded in four guiding principles, namely: the commitment of countries, the integrated strategy of successful initiatives, the appropriate use of digital health technologies, and the resolution of key challenges in less developed countries through the implementation of digital health technologies (1).

Several poor and developing countries, such as Brazil and India, have proposed their own strategies that align with the principles outlined by the WHO (2, 3). They also anticipate a series of actions in the coming years aimed at monitoring people's health through digital health, thereby enhancing access to data and reducing the cost of services, among other advantages.

Among the numerous health problems encountered in different countries, limitations resulting from decreased or loss of motor activity hold significant prominence, as they can lead to serious consequences, even in rich countries (4–6). Falls and loss of mobility can be key factors contributing to a decline in the quality of life, particularly in the older age group (7–9). Conditions such as Parkinson's disease, Alzheimer's disease, arthritis, diabetes, obesity, sedentary lifestyle, among others, may result in some degree of loss of motor functionality, thereby creating a demographic that will seek healthcare services, either private or public, in their respective countries (10–12).

The gold standard methods for motor assessment, in general, are systems such as electromyography, force platforms, and video capture systems (13–15). These methods are high-cost and are not readily available to the general population even in rich countries. In developing or impoverished nations, these methods are often inaccessible even to the wealthier individuals, as they are typically restricted to research centers or specialized referral facilities. Motor assessments in populations prone to motor functional loss in these regions are frequently conducted using functional scales (16–18). However, these scales may not always allow for proper monitoring of functional losses or only enable the identification of the existence of a problem without facilitating a graded assessment of the extent of the problem (19–23).

Especially in the last two decades, numerous proposals for motor assessment have been proposed and validated using sensors present in smartphones (21, 22, 24, 25), thereby opening a range of opportunities for access by individuals, particularly those with lower economic means, to appropriately monitor their motor condition. This development equips healthcare professionals with tools to implement the most effective therapeutic interventions.

## Motor assessment using smartphones

Smartphones contain an array of sensors with various functionalities that enable the monitoring of the device's position, video and sound capture, brightness, temperature, among other pieces of information (26). Programmers can develop applications, commonly known as apps, specifically designed for smartphones, which read data from these sensors and utilize this information for a particular task.

**Figure 1:**
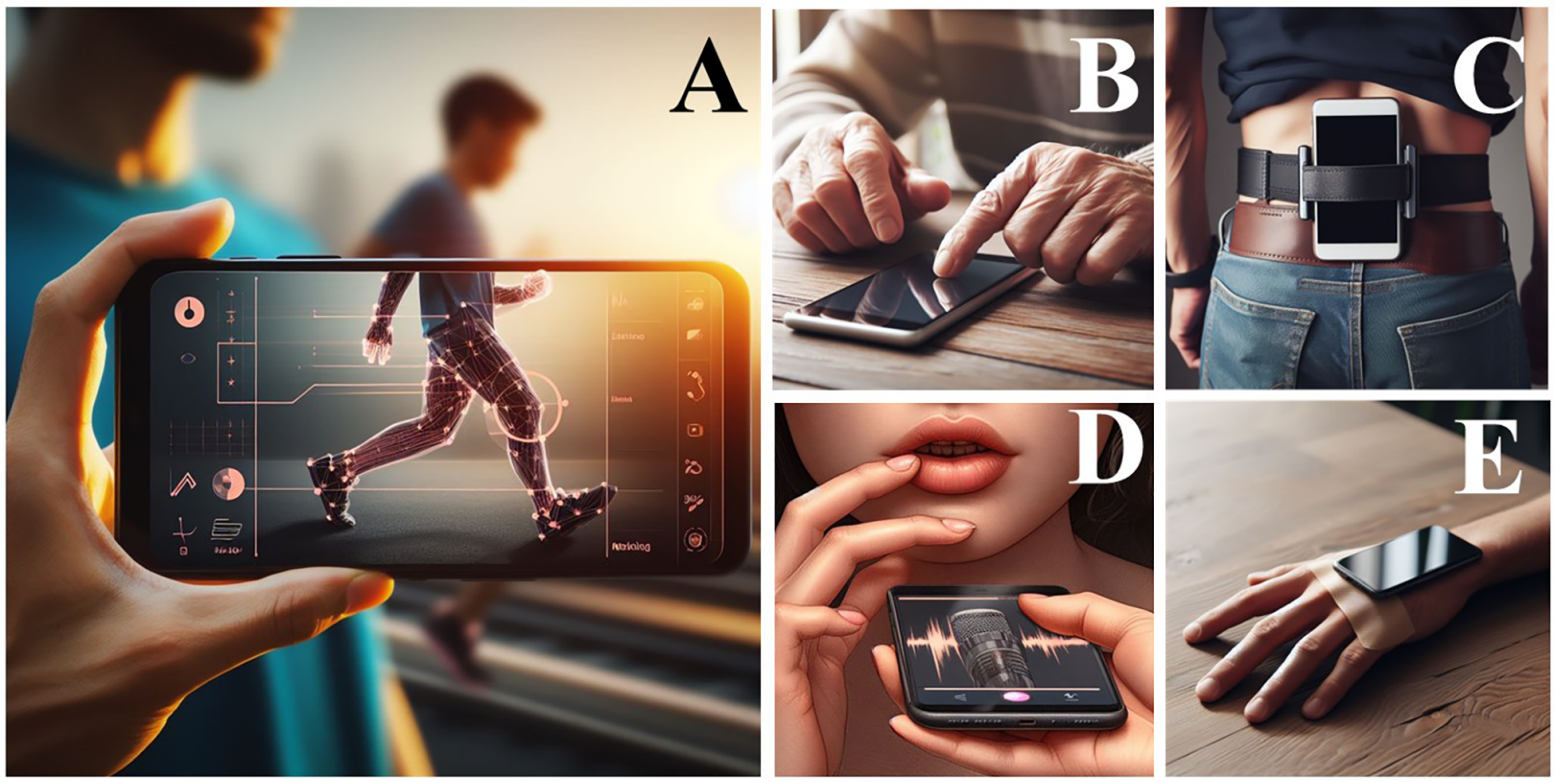
Examples of smartphone uses to evaluate the motor functions such as walk using video capture methods (**A**), using finger tapping test using touchscreen capture (**B**), using inertial sensors for static balance control and mobility evaluation while held in a body segment (**C**), using microphone to record the voice (**D**), and inertial sensors to evaluate resting handing tremor. Images created using artificial intelligence (see Supplementary material for prompts to generate figures).

In the field of healthcare, numerous applications for assessment, monitoring, and intervention are continually proposed and released in app stores each day. Following this trend, many applications have been developed for various motor assessment tasks, and various studies have been undertaken to evaluate the validity of these applications for the assessment of the motor tasks they aim to perform (Figure 1).

Inertial sensors such as accelerometers and gyroscopes have been utilized to assess balance, mobility, tremor, and levels of physical activity in various healthy or diseased populations (27–30). Typically, these analyses involve affixing the smartphone to the bodies of individuals to be tested, aiming to record linear (acceleration) or rotational (angular velocity) changes in the body segment-smartphone combination. Various analyses can be conducted on the inertial time series to extract features that may serve as biomarkers for the assessed motor functionality (31, 32).

The touchscreen of smartphones has been employed to assess the hand's movement and motor coordination in patients, as seen in tasks such as the finger tapping test and drawing on the Archimedean spiral (24, 32–34). Information regarding touches allows for determining the frequency of finger taps on the screen within a specified time interval, the duration between taps, the locations of the taps, the similarity between the patient's drawing and the presented model, and these characteristics can be used to characterize whether the tested individuals fall within an expected pattern for clinically healthy individuals of their respective age groups (34).

Video and audio capture are also strategies for gathering biological information using the camera or microphone of the smartphone (25, 21). Specific algorithms for video and sound analysis have been made available and validated in populations with various diseases. In general, the examples described here do not require the latest-generation smartphones, and depending on the complexity of the task, data processing may need to be performed in the cloud, necessitating an internet connection.

## Motor assessment based on smartphones and public health

The advantages of using smartphones for motor assessment in public healthcare services of developing countries with significant social inequalities, such as Brazil and India, may include:

- Access to an objective motor assessment tool: Smartphone applications have been considered as a low-cost and valid tool for disease screening (35). The cost of gold standard methods for motor assessment is high, and access to them is challenging. Research centers employing these gold standard methods have their own research interests, limiting the clientele that can be served. Reference centers often cannot accommodate all those in need of qualified assessment, frequently having to prioritize the most severe cases for evaluation.
- Waiting time for assessments: Smartphones are widely distributed globally, with an average of approximately one device per inhabitant worldwide. If primary healthcare professionals utilized smartphones for monitoring motor functionality in patients, the majority of the population in need of some form of assessment could be served at the primary care level, reducing the waiting time for evaluations at specialized centers.
- Costs for public or private healthcare systems: It is conceivable that the routine use of smartphones for motor assessment of patients could aid in the early identification of motor functional losses, enabling interventions to prevent major complications or even reverse the patient's clinical condition. Falls and immobility among patients cost billions of dollars annually for both individuals and healthcare systems in different countries. However, it is in developing or impoverished countries where these expenses are most keenly felt, given that funding for monitoring programs for various diseases is more limited than in rich countries. Additionally, there will be a reduced need to purchase gold standard equipment for different services.
- Improved quality of life for individuals: Identifying motor functional losses and early intervention to mitigate such losses increases the likelihood of individuals becoming less ill and experiencing an enhanced quality of life.

## Limitations and considerations for digital interventions using smartphones for motor evaluation

It is important to emphasize that, despite the advantages digital interventions can offer in healthcare and service delivery, it does not endorse the substitution of traditional models, but an alternative way of intervention. Despite all the described advantages, certain considerations must be taken into account before initiating a digital health intervention to implement motor assessments using smartphones.

Firstly, it is crucial to recognize that the use of smartphones for motor assessment should not be interpreted as a substitute for gold standard methods of movement analysis (36). Instead, they should be viewed as additional tools that allow for meeting a greater demand in primary healthcare. In cases of detecting more severe functional losses, individuals should be referred to specialized services for a more accurate diagnosis.

It is not entirely clear whether all developed protocols for motor functional assessment can be performed as self-tests by the patients themselves or with the assistance of a healthcare professional (37). Some studies have aimed to assess the reliability of self-testing, but further research involving larger and diverse populations is needed to reach a consensus (37, 38). We understand that for now, the apps should be used by professionals to safeguard correct assessment until more evidence is available.

Another point for healthcare managers to consider is whether the smartphone used in the service will be provided by the institution or if it will be the professional's personal device. Using the professional's smartphone may offer an advantage as the service wouldn't need to purchase the device. However, patient assessment data would be on the private property of the professionals. Purchasing smartphones exclusively for motor assessment use could represent an additional cost for the service, albeit far less than the cost required to acquire gold standard motion analysis equipment.

Another factor that slows down the integration of smartphones into movement assessment in primary care services is that many of the apps are still somewhat experimental, and their analyses require the use of offline programming routines for analysis. Moreover, even in apps that perform analyses on the data, few provide normative population values for various analysis parameters (39). There are also few apps with assessment protocols that allow for a more comprehensive characterization of motor functionality, requiring the downloading of multiple apps for different assessments, potentially making patient health monitoring somewhat more complex.

Finally, there's the issue of adequately training healthcare professionals who would conduct motor assessments using smartphones. Some expenditure would be necessary for the training and skill development of these professionals in the use of digital tools applied to patient assessments.

## Considerations for the use of artificial inteligence in apps for motor evaluation

In human movement assessment applications, it is possible to extract an immense amount of characteristics from the motor task being evaluated. There is a consensus that artificial intelligence algorithms can better handle this multi-dimensionality of data and capture complex relationships between variables, even in the presence of non-linearities, reducing the need for human intervention in the decision-making process to determine whether the assessment is considered normal or altered (40).

At present, the use of artificial intelligence in motor assessment applications is not yet a reality, and this is due to various reasons, but among them, we consider some to be important when addressing public health in developing countries:

(i) Database: Developers would need to create a large database of healthy individuals, taking into account demographic characteristics such as age and gender, as well as a database of individuals with different diseases and varying degrees of motor impairment. All of this not only takes time, but can be very costly for developers. Databases with small samples may not generalize adequately to a large population.
(ii) Internet availability to users: Some machine learning algorithms may need to run in the cloud, consuming the user's internet data plan. Especially in poorer countries, this can be a major issue, as network coverage is not extensive enough to reach all cities.

## Conclusion

There are numerous advantages to using smartphones in motor assessment that span from the well-being of patients to the costs borne by governments for the health of the population. However, the digital health intervention process for motor assessment should be carefully planned to ensure that a strategy with significant potential can be effective compared to the current applied models.

Developing and impoverished countries should seriously consider the possibility of using smartphones to monitor the motor functionality of the population as part of public policies. Perhaps it would be prudent to initially test these interventions in small services, hospitals, or communities and evaluate the different effects before progressing to larger segments of the population.

## Data Availability

The original contributions presented in the study are included in the article/**Supplementary Material**, further inquiries can be directed to the corresponding author.
