# Supplementary material for: Enhancing public health in developing nations through smartphone-based motor assessment
Source: Front Digit Health. 2024 May 21;6:1345562. doi: 10.3389/fdgth.2024.1345562 (PMC11148357; doi:10.3389/fdgth.2024.1345562)
Supplement: Supplementary file 1 [file Datasheet1.docx]

SUPPLEMENTARY MATERIAL

Figure 1 was created using DALL-E from Bing Image Creator. The prompt for each panel were written in Portuguese as follow:

Panel A: “Mostra a tela de um smartphone filmando uma pessoa andando com marcações de análise do movimento”

Panel B: “Mão idosa digitando a tela sensível ao toque de um smartphone sobre uma mesa”

Panel C: “Alguém em pé com smartphone preso as costas na região lombar por uma cinta na cintura”

Panel D: “Desenho realista de uma mão segundo um smartphone perto da boca de uma segunda pessoa para gravar a voz dessa pessoa com o microfone do próprio smartphone. Na tela do smartphone coloque arte de ondas sonoras. Por favor não mostre microfone na imagem”

Panel E: “Uma mão repousando para fora de uma mesa com um smartphone preso por fita dupla face sobre a sua porção dorsal”
